# Nano-Radiopharmaceuticals in Colon Cancer: Current Applications, Challenges, and Future Directions

**DOI:** 10.3390/ph18020257

**Published:** 2025-02-14

**Authors:** Ajnas Alkatheeri, Suliman Salih, Noon Kamil, Sara Alnuaimi, Memona Abuzar, Shahd Shehadeh Abdelrahman

**Affiliations:** 1 Department of Radiography and Medical Imaging, Fatima College of Health Sciences, Abu Dhabi 3798, United Arab Emirates; suliman.salih@fchs.ac.ae; 2 National Cancer Institute, University of Gezira, Wad Madani 2667, Sudan; 3 Department of Pharmacy, Fatima College of Health Sciences, Abu Dhabi 3798, United Arab Emirates; noon.kamil@fchs.ac.ae (N.K.); sara.alnuaimi@fchs.ac.ae (S.A.); memona.abuzar@fchs.ac.ae (M.A.); 4 College of Medicine and Health Sciences, Khalifa University, Abu Dhabi 3798, United Arab Emirates; shahd.shehadeh.2897@gmail.com

**Keywords:** colorectal cancer, nano-radiopharmaceuticals, nanoparticles, radiolabeled, theranostics

## Abstract

Colon cancer remains a significant global health challenge; however, the treatment outcome for colon patients can be improved through early detection and effective treatment. Nano-radiopharmaceuticals, combining nanotechnology with radiopharmaceuticals, are emerging as a revolutionary approach in both colon cancer diagnostic imaging and therapy, playing a significant role in the management of colon cancer patients. This review examines the use of nano-radiopharmaceuticals in the diagnosis and treatment of colon cancer, highlighting current applications, challenges, and future directions. Nanocarriers of radionuclides have shown potential in improving cancer treatment, including liposomes, microparticles, nanoparticles, micelles, dendrimers, and hydrogels, which are approved by the FDA. These nanocarriers can deliver targeted drugs into malignant cells without affecting normal cells, reducing side effects. Antibody-guided systemic radionuclide-targeted therapy has shown potential for treating cancer. Novel cancer nanomedicines, like Hensify and 32P BioSilicon, are under clinical development for targeted radiation delivery in percutaneous intratumoral injections. Although using nano-radiopharmaceuticals is a superior technique for diagnosing and treating colon cancer, there are limitations and challenges, such as the unintentional accumulation of nanoparticles in healthy tissues, which leads to toxicity due to biodistribution issues, as well as high manufacturing costs that limit their availability for patients. However, the future direction is moving toward providing more precise radiopharmaceuticals, which is crucial for enhancing the diagnosis and treatment of colon cancer and reducing production costs.

## 1. Introduction

One of the major global challenges in managing and curing colon cancer is early detection and providing effective and efficient treatment methods. To achieve this goal, numerous developments have successfully used nanotechnology combined with radiopharmaceuticals, which has made personalized medicine for colon cancer a possible future direction. Nano-radiopharmaceuticals have emerged as a promising approach for the diagnosis and treatment of cancer in the past three decades. Nanoparticles utilized and investigated in biological applications encompass synthetic lipid-based structures such as liposomes, micelles, uni- and multilamellar vesicles, and exosomes, alongside highly organized polymers, dendrimers, and metal nanoparticles. The developed radionuclides and nano-radiopharmaceuticals play a significant role in enhancing accuracy, affectivity, resolution and targeted imaging, and medication delivery, overcoming some conventional methods [1,2,3].

In recent decades, radionuclides that release particulate radiation, such as α, β, protons, neutrons, and/or magnetic waves such as X-rays and γ-rays, have been used for the diagnosis and treatment of many types of cancers, including colon cancer. Radionuclides can act as therapeutic agents by destroying and eliminating the targeted cancer cells. They can also act as diagnostic agents when the emitted gamma ray or annihilation gamma ray pairs are detected by detector arrays positioned around the patient to be imaged. The FDA has approved a number of radionuclide imaging and/or therapeutic agents, which are currently being used extensively in clinical settings for a variety of purposes [3]. Nanoparticles possess unique features such as small size, diverse shapes, high sensitivity, and specific chemical compositions depending on their nano-formulations. These properties help them to work as contrast agents and coating materials in imaging for cancer diagnosis, as well as therapeutic agents by delivering nano-drugs. Additionally, they enhance the biochemical and physiological understanding of colon cancer, making them promising novel tools for colorectal cancer management. However, numerous challenges face the development of nanoparticles due to their complex pharmacokinetics [3,4,5].

Recent advancements in colon cancer treatment have introduced novel therapies that greatly enhance patient outcomes. Immunotherapy has shown promising results in improving survival rates in patients with advanced stages of colon cancer, especially with immune checkpoint inhibitor targeting. Additionally, due to the heterogeneity of colon cancer, personalized medicine has emerged as a noteworthy advancement in its treatment because it considers the genomic profile and associated biomarkers of the disease. One of the new advanced approaches used to treat CRC, which depends on the tumor microenvironment (TME), is using the unique physiological structure and metabolic characteristics of Veillonella atypica (VA) as a carrier to load Staphylococcus aureus cell membrane-coated BaTiO_3_ nanocubes (SAM@BTO), which increases the bioavailability by achieving cascade targeting of the formulation and mediating highly effective synergistic tumor catalysis and immune therapy through the effective killing of the primary tumor and regulation of the immunosuppressive microenvironment, achieving synergistic catalytic and immune treatment of CRC under in vitro ultrasonic stimulation [4,5,6]. Another method is using CaO_2_ NPs to act as an in vivo switch for self-triggering thermoelectric dynamic therapy by applying the thermoelectric material BST using Bi(NO_3_)_3_·5H_2_O, Na_2_TeO_3_, and SbCl_3_ as substrates and PVP as a blocker in an ethylene glycol solution. Bi0.5Sb1.5Te3 nanosheets (BST NSs) have been found to have the highest thermoelectric conversion efficiency at very low temperatures, making them ideal for biomedical applications in vivo. However, CaO_2_ NPs also have the potential to act as an in vivo switch for self-triggering thermoelectric dynamic therapy due to their tumor microenvironment (low pH)-specific water liberation thermal effect, which has not yet been explored. Tumor-specific self-triggered thermoelectric nanoheterojunction combined catalytic therapy, ion interference therapy, and immunotherapy exhibits excellent antitumor performance in female mice [7]. Addressing these limitations requires further research and investigation into nanoparticle design and radiolabeling clinical application approaches. Future developments in nano-radiopharmaceuticals for colon cancer are expected to focus on improving the biocompatibility and targeting efficiency of these agents. Advances in personalized nanomedicine, which tailors treatments based on individual patient profiles, and the integration of new imaging technologies with nanomedicine hold promise for enhancing both diagnostic and therapeutic outcomes [8,9].

This review aims to provide a description of the nano-radiopharmaceuticals that are used in colon cancer imaging and therapy, nanocarriers for the targeted delivery of radionuclides, and nano-targeted radiopharmaceuticals for tumor diagnosis and therapeutics, as well as their current applications, challenges, and future directions.

## 2. Common Properties of Nanoparticles and Radionuclides Used for the Diagnosis and Treatment of Colon Cancer

**Figure 1:**
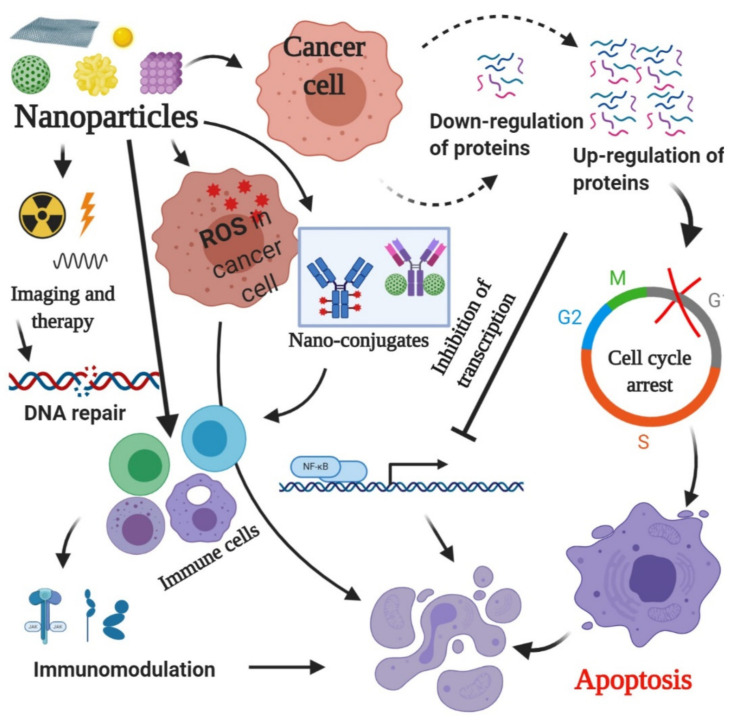
The image elucidates a multifaceted approach to cancer therapy using nanoparticles. These particles engage with cancer cells by generating reactive oxygen species, affecting protein expression and triggering cell cycle arrest and apoptosis. Additionally, they can enhance the immune response, making them a promising tool in cancer treatment strategies [13].

Nano-pharmacology is concerned with the application of nanotechnology based on novel pharmacological principles to increase therapeutic efficacy, reduce side effects, and achieve the targeted delivery of medicine to specific sites in a controlled manner (Figure 1). Since the advent of nanotechnology over four decades ago, the foundations for nanotechnology applications for both diagnostic and therapeutic purposes have been laid out in a very precise manner for its use [10,11]. There are many applications of nanoparticles in the screening, detection, and treatment of colorectal cancer [12].

### 2.1. Properties of Common Nanoparticles Used in Colon Cancer Management

**Figure 2:**
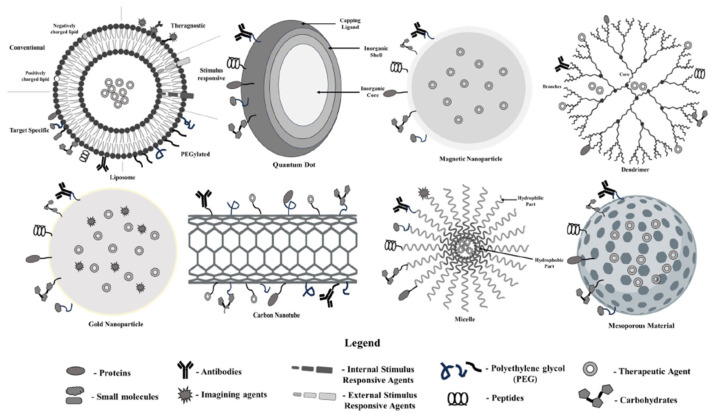
Common nanoparticles used in colon cancer management; each type has a unique shape and strucutre help it to cary various legnads and deliver nano-radiopharmacetuicals [14].

Different types of nanoparticles and nanotools are commonly used for colon cancer management (Figure 2). Usually, these nanoparticles are used for the delivery of nano-radiopharmaceuticals. The following section enlists the properties of nanotools for the delivery of nano-radiopharmaceuticals.

#### 2.1.1. Properties of Nanocrystal Quantum Dots (QDs)

Quantum dots (QDs) are semiconductor nanoparticles with unique properties attributed to their quantum confinement effect, which arises from their small size (typically 2–10 nanometers). This effect results in discrete energy levels and size-dependent optical properties, allowing QDs to emit light across a broad range of wavelengths. The emission wavelength of QDs is directly correlated with their size, with smaller QDs emitting higher-energy light (e.g., blue), and larger QDs emitting lower-energy light (e.g., red). This tunability makes them highly advantageous for applications in bioimaging and diagnostics [13].

Alongside their adjustable properties, QDs exhibit enhanced photostability and brightness when compared to conventional fluorescent dyes, which often experience photobleaching after prolonged light exposure. The ability to resist photobleaching renders QDs ideal for extended applications, including ongoing monitoring in immunotherapy, real-time imaging, and multimodal imaging systems. Their elevated surface-to-volume ratio enables effective functionalization with various biomolecules, including peptides, antibodies, and other targeting agents, thereby enhancing precise drug delivery, imaging, and multimodal diagnostics in biological systems.

However, while many of the most recent studies on QDs focus on minimizing the toxicity associated with heavy metals such as cadmium in bioimaging applications, it is important to note that many non-toxic, surface-modified, and highly bright QDs are now being developed. The non-toxic alternatives, featuring optimized surface modifications like biocompatible coatings, demonstrate potential for improving the stability, imaging brightness, and functionalization of QDs, positioning them as promising candidates for cancer cell imaging and various clinical applications. This developing field is essential for promoting the safe application of QDs in clinical environments while preserving their beneficial optical characteristics, presenting opportunities for early diagnosis and targeted treatment with reduced toxicity [15,16,17,18,19].

#### 2.1.2. Properties of Iron Oxide Nano-Formulation Nanoparticles

Iron oxide nanoparticles (IONPs), particularly in the form of magnetite (Fe_3_O_4_) or maghemite (γ-Fe_2_O_3_), have gained significant attention in biomedical applications due to their unique magnetic properties, biocompatibility, and ease of surface modification. These nanoparticles are superparamagnetic, which means they exhibit magnetic behavior only in the presence of an external magnetic field and show no residual magnetism once the field is removed. This property makes them ideal for use in magnetic resonance imaging (MRI) as contrast agents, drug delivery systems, and magnetic hyperthermia treatments for cancer [20].

IONPs can be functionalized with various polymers, proteins, or antibodies, allowing for targeted therapeutic and diagnostic applications. Their small size (typically less than 100 nm) enhances cellular uptake, facilitating targeted drug delivery, especially in tumor tissues through the enhanced permeability and retention (EPR) effect [21]. Furthermore, their surface can be engineered to reduce toxicity and improve circulation time, making them suitable for in vivo applications [22].

However, concerns regarding their potential cytotoxicity and long-term biocompatibility persist, as iron ions released from these particles can lead to oxidative stress in cells. Ongoing research focuses on improving the safety and efficacy of iron oxide formulations while expanding their applications in theranostics—combining therapy and diagnostics in a single platform [23].

#### 2.1.3. Properties of Poly Lactic-co-Glycolic Acid (PLGA) Nanoparticles

Poly Lactic-co-Glycolic Acid (PLGA) nanoparticles are widely used in drug delivery systems due to their biodegradability, biocompatibility, and ability to encapsulate a wide range of therapeutic agents, including small molecules, peptides, proteins, and nucleic acids. PLGA is an FDA-approved copolymer composed of lactic acid and glycolic acid, which degrade into non-toxic by-products (carbon dioxide and water) through hydrolysis within the body. The rate of degradation can be controlled by adjusting the ratio of lactic acid to glycolic acid in the polymer chain, offering flexibility in tailoring the release kinetics of encapsulated drugs [24].

The versatility of PLGA nanoparticles lies in their ability to be surface-functionalized with targeting ligands, which improves their ability to deliver therapeutic agents to specific tissues or cells, particularly in cancer therapy [25]. Due to their nanoscale size (typically between 100 and 300 nm), they exhibit enhanced permeability and retention (EPR) effects, allowing them to accumulate more efficiently in tumor tissues. This enhances their potential for passive targeting in cancer treatment, and their ability to cross biological barriers further amplifies their utility in drug delivery [26].

Additionally, PLGA nanoparticles can protect encapsulated drugs from premature degradation, improving their stability and bioavailability. They are also recognized for their controlled and sustained release profile, which minimizes the need for repeated doses, improving patient compliance and therapeutic efficacy [27].

#### 2.1.4. Properties of Carbon Nanotube (CNT) Nanoparticles

Carbon nanotubes (CNTs) are cylindrical nanostructures made from rolled-up sheets of graphene, with unique physicochemical properties that make them valuable for various biomedical applications, including drug delivery, imaging, and cancer therapy. Their exceptional mechanical strength, large surface area, and high electrical and thermal conductivity are some of their defining characteristics [28]. CNTs can be single-walled (SWCNTs) or multi-walled (MWCNTs), depending on the number of graphene layers involved, and they possess diameters ranging from less than 1 nm to several tens of nanometers.

One of the most significant properties of CNTs is their ability to be functionalized, allowing for the attachment of various molecules such as drugs, proteins, or DNA. This property is crucial in enhancing the biocompatibility and solubility of CNTs in aqueous environments, which otherwise tend to be hydrophobic [29]. Their hollow structure enables the encapsulation of therapeutic agents, while their nanoscale size facilitates penetration into biological tissues, making them highly effective in targeted drug delivery systems.

Moreover, CNTs exhibit strong photothermal conversion abilities, meaning they can convert near-infrared light into heat. This property is particularly useful in cancer treatment, where CNTs can be employed to ablate cancerous cells via hyperthermia [30]. Additionally, CNTs have been used in diagnostic applications as contrast agents in imaging modalities such as MRI due to their ability to carry gadolinium or other contrast-enhancing agents.

However, their use is also limited by concerns over toxicity and biocompatibility, as some studies have indicated that CNTs can induce inflammatory responses or cytotoxicity under certain conditions [31]. As such, there is ongoing research into improving the functionalization and safety profiles of CNTs for broader clinical applications.

#### 2.1.5. Properties of Dendrimer Nanoparticles

Dendrimers are highly branched, tree-like nanostructures with unique properties that make them suitable for various biomedical applications, including drug delivery, imaging, and gene therapy. The key feature of dendrimers is their well-defined, monodisperse, and highly branched structure, which allows for precise control over their size and surface functionality [32]. This branching creates numerous functional groups on the surface, enabling the attachment of drugs, targeting ligands, or imaging agents, making them highly adaptable for specific medical needs.

Their high degree of functionality enhances their solubility and biocompatibility, particularly in aqueous environments, which is crucial for biological applications. The internal cavities of dendrimers can encapsulate small molecules, protecting them from degradation until they reach their target. Moreover, their nanoscale size (typically ranging from 1 to 10 nm) facilitates efficient cellular uptake, making them effective carriers for therapeutic agents [33].

Additionally, dendrimers have been extensively studied for their use in targeted cancer therapy due to their ability to passively accumulate in tumor tissues through the enhanced permeability and retention (EPR) effect. However, their toxicity and immunogenicity are still areas of ongoing research, as modifications to their surface chemistry may alter their interaction with biological systems [34].

#### 2.1.6. Properties of Liposome Nanoparticles

Niosomes and bilosomes represent two more novel carriers coming into focus in targeted drug delivery systems due to their unique structural and functional features. Niosomes are nonionic surfactant vesicles that entrap both hydrophilic and hydrophobic drugs, offering better stability and controlled release under physiological conditions [35,36]. Being highly biocompatible with lower toxicity, they enhance the bioavailability of drugs and, as such, offer great promise in targeting tissues or cells at particular sites. Similarly, bilosomes are modified niosomes that have been stabilized with bile salts, thus improving the oral bioavailability of drugs and vaccines. Bile salts protect against gastrointestinal degradation and absorption through the intestinal wall, and thus bilosomes are very important in the oral delivery of peptides and proteins [37,38]. Both of these carriers are gaining much attention because they have the potential to enhance therapeutic efficacy while minimizing off-target effects [39].

Liposomes are versatile nanoparticles characterized by a spherical structure composed of one or more lipid bilayers surrounding an aqueous core. This unique architecture allows them to encapsulate both hydrophilic and hydrophobic molecules, making them highly adaptable for drug delivery applications. Hydrophilic drugs are stored in the aqueous core, while hydrophobic drugs can be integrated into the lipid bilayer [40]. Liposomes exhibit excellent biocompatibility due to their lipid composition, which mimics natural cell membranes, resulting in minimal toxicity and enhanced drug bioavailability. Moreover, their size and surface properties can be tailored to improve pharmacokinetics and control drug release rates, thereby reducing side effects and increasing therapeutic efficacy [41].

A key feature of liposomes is their potential for targeted drug delivery. Functionalization with ligands such as antibodies or peptides can direct liposomes to specific tissues or cells, particularly in cancer therapy, where they can deliver cytotoxic drugs directly to tumor sites, minimizing damage to healthy tissues. Liposomes are also used in imaging techniques, as they can encapsulate contrast agents, enhancing diagnostic imaging modalities such as MRI or CT. Despite their advantages, challenges like instability, rapid clearance by the immune system, and the need for further optimization in large-scale production remain hurdles for clinical applications [42].

#### 2.1.7. Properties of Gold Nanoparticles (AuNPs)

Among the noble metals, gold nanoparticles (AuNPs) are regarded as the most stable nanomaterials, extensively utilized for synthesizing nanostructures with diverse forms, such as nanocubes, nanospheres, nanorods, nanoflowers, nanobranches, nanowires, nano-bipyramids, nanoshells, and nanocages [43,44]. Technological advancements have enabled precise surface coating of AuNPs with specific shapes and sizes. These properties enhance the safety and specificity of gold nanomaterials, making them effective agents for anticancer therapy and drug delivery [45,46,47]. Additionally, studies utilizing artificial intelligence and mathematical modeling suggest that AuNPs can adjust their optical densities, light absorbance, and wavelengths, with optimal wavelength tuning and nanoparticle size increasing light absorption, thereby boosting the effectiveness of AuNPs against cancer cells [48]. In a recent study, AuNPs were found to improve cisplatin delivery and enhance chemotherapy efficacy by decompressing colorectal cancer (CRC) vessels [49]. Furthermore, AuNPs, when combined with nucleic acids, have been employed to facilitate cellular uptake at the molecular level [50].

#### 2.1.8. Properties of Core/Shell Polymeric Nano-Formulations

There has been a growing focus on the development of core/shell nanoparticles made from two or more materials [51,52]. These nanoparticles exhibit a core/shell configuration, wherein the atoms on the surface differ from those inside the core. The surface chemistry of core/shell nanomaterials is typically analyzed using several advanced techniques, including secondary ion mass spectrometry and X-ray photoelectron spectroscopy [53,54]. Various combinations of materials can be employed in core/shell nanoparticles, such as organic/organic, organic/inorganic, and inorganic/inorganic, depending on the intended application [55]. These configurations are specifically designed to enhance the stability, functionality, and dispersibility of the core. Additionally, these particles can enable controlled release mechanisms, reducing the consumption of expensive materials [53]. Core/shell particles have significant biomedical applications, including bioimaging for cell labeling, targeted drug delivery, and tissue engineering [56,57,58].

#### 2.1.9. Properties of Mesoporous Nanoparticles

Mesoporous nanoparticles, with their high surface area, porosity, chemical stability, and biocompatibility, are ideal for drug delivery, imaging, and catalysis. They can be tuned to accommodate different sizes and shapes, allowing for controlled release. These nanoparticles also exhibit optical transparency and magnetic properties, making them suitable for imaging and targeting applications. They also have high thermal stability and mechanical strength, making them suitable for applications requiring resistance to mechanical stress or abrasion. Their unique electrical properties, such as conductivity or semiconductivity, make them suitable for various applications. Liu et al.’s study on mesoporous litchi-shaped nanoparticles showed controlled release of chemotherapy medications like DOX, potentially enhancing the antitumor efficacy of radiotherapy by increasing X-ray absorption in tumor tissues. Further research is needed to fully explore these properties and applications [59,60].

### 2.2. Properties of Radionuclides as Part of Nano-Radiopharmaceuticals

Radionuclides in nano-radiopharmaceuticals possess unique properties that make them useful in both diagnostic and therapeutic applications in colon cancer. The properties of these agents include their physical half-life, the type of radiation they emit, the energy of their particles, and their ability to be conjugated with specific targeting molecules, such as antibodies or peptides. The half-life of a radionuclide plays an important role in determining how long it remains active in the body. It is often preferred to use radionuclides with a suitable short half-life for imaging purposes to minimize radiation exposure to the patient, whereas therapeutic radionuclides are often used to ensure a sufficient radiation dose is delivered to the tumor over time (Table 1) [61,62]. For isotopes used in radiation therapy, the optimal half-life range is often between 6 h and 7 days. This range minimizes damage to healthy tissues while enabling effective treatment.

Radiopharmaceuticals that generate either positrons or gamma radiation to locate microorganisms are preferable for diagnostic applications since they have shorter half-lives. On the other hand, longer half-lives are necessary for therapeutic radiopharmaceuticals to provide the target site with an adequate radiation dose (Table 2) [63,64].

**Type of Radiation Emitted**: A diagnostic radionuclide emits gamma rays or positrons that can be detected by imaging devices such as PET scans (Positron Emission Tomography) or SPECT scans (Single-Photon Emission Computed Tomography). On the other hand, therapeutic radionuclides emit alpha or beta particles, which have higher linear energy transfer and short penetration in cancer tissues [68].

**Radiation Energy of Emitted Particles**: Both imaging clarity and therapeutic efficacy depend on the radiation energy level emitted. Generally, high-energy gamma rays are best suited to penetrating tissues and providing clear imaging, while lower-energy particles are more appropriate for localized tumor treatment since they cause less damage to surrounding healthy tissues [62,69].

**Targeting Specificity**: In colon cancer therapy, radionuclides are often attached to molecules that target cancer cells specifically, such as monoclonal antibodies. As a result of this targeting ability, radionuclides accumulate in the tumor, maximizing the therapeutic effect while minimizing the negative effects on healthy tissue [70].

As a result of these properties, nano-radiopharmaceuticals can be used in both the diagnosis and treatment of colon cancer, enabling precise imaging of the disease as well as targeted treatment.

## 3. Clinical Application of Nano-Radiopharmaceuticals in Colon Cancer

Cancer nanotechnology has transformed the current treatment systems because it provides more efficient cancer diagnostics and therapeutics. It can be used in cancer detection at early stages and to deliver anticancer drugs to malignant cells [71,72,73,74,75,76].

Nanocarriers of radionuclides have been proved to improve the pharmacokinetics and therapeutic efficacy of cancer treatments. They include liposomes, microparticles, nanoparticles, micelles, dendrimers, and hydrogels. Liposomes and albumin nanoparticles were approved by the US FDA for clinical practices as therapeutic carries. Customized nanoscale constructs can serve as targeted drug delivery vehicles to deliver radionuclide agents in large doses into malignant cells without affecting normal cells, which reduces the side effects compared with many current cancer therapies [73,74,75,76,77,78,79].

Antibody-guided systemic radionuclide-targeted therapy has great potential for treating cancer, as exemplified by the FDA approval of two radiolabeled anti-CD20 monoclonal antibodies, 90Y-ibritumomab (Zevalin) and 131-I tositumomab (Bexxar), in 2002 and 2003, respectively. Several novel cancer nanomedicines reached the market in the 2010s. For example, Hensify, composed of a crystalline hafnium oxide (HfO2) core and amorphous thin biocompatible coating, acts as a radiosensitizer. It absorbs external radiotherapy and enhances its action through the generation of a localized radiation dose. Many cancer radionuclide nanomedicines, such as 32P BioSilicon, are currently under clinical development. 32P BioSilicon is a porous silicon. nanoparticle containing radioactive phosphorus (32P), developed by BioSilicon™. 32P is ideal for radiotherapy because it is a pure β-particle emitter. 32P BioSilicon’s route of administration is percutaneous intratumoral injection, which provides targeted radiation for the malignant cells [76,80].

### 3.1. Nano-Radiopharmaceuticals and Tracers for Colon Cancer Diagnosis

Radiopharmaceuticals are essential in tumor diagnostics, with pharmaceutical radionuclides frequently attached to specific targeting molecules, like peptides, antibodies, or small molecules. These compounds stand out due to their capacity to attach to indicators present in cancer cells. A lot of progress has been made in the study of colorectal cancer by using radiopharmaceutical tracers like 18 FDG to target specific receptors on cancer cells or to focus on parts of the tumor that have a lot of metabolic activity. However, for a considerable period, researchers have extensively utilized options like FAZA and 11C Choline. For example, FAZA is useful for seeing tumors that do not have enough oxygen, while 11C Choline is useful for seeing prostate cancer and other cancers that have changed choline metabolism. The different choices make radiopharmaceuticals better at diagnosing, giving doctors more tools for finding tumors and checking how active they are [81,82,83].

#### 3.1.1. Combination of Nanoparticles and 18F-FDG in Colon Cancer Theranostics

Theranostics is an innovative approach in the oncology discipline that integrates the diagnosis and treatment of neoplasms. In the case of colon cancer, this method enables clinicians to visualize tumor sites and determine the effectiveness of therapy in real time. For this purpose, the inclusion of nanoparticles along with 18F-FDG is an innovative approach that enhances the diagnosis realistically and provides therapeutic benefits. Nanoparticles also have the ability to carry 18F-FDG molecules toward the tumor tissue, thereby increasing their imaging and treatment effectiveness [83].

18F-fluorodeoxyglucose (FDG) has emerged as the most predominant radiopharmaceutical for PET examination, which depicts the cancer cells based on their highly pronounced uptake of glucose, a characteristic of malignant cells due to the Warburg effect [82]. However, its function in theranostics is not only limited to imaging. Within multifunctional systems, when used with nanoparticles, 18F-FDG can be utilized for tumor imaging and therapeutic delivery, improving the treatment efficacy of colon cancer. The combination maximizes the sensitivity and specificity of tumor detection as well as the assessment of treatment efficacy in one patient over time [84].

##### Nanoparticles and 18F-FDG as a Functionalized Multi-Carrier

Nanoparticles, with their nanometer size and their surface functional groups, are ideal for theranostic procedures. This approach also opens up the possibility of creating lighter, shallower designs. By embedding 18F-FDG into these tumor core biosystems, the cancerous cells are killed by chemotherapeutic or radiation sensitizers targeted to the tumor location. In colon cancer theranostics, nanoparticles coated with antibodies or peptides that target tumors increase the in vitro retention of 18F-FDG by cancer cells while reducing normal cellular retention. Through this targeted delivery system, the radiotracer and drug are delivered at a higher concentration to the tumor, thereby optimizing both imaging accuracy and therapeutic effectiveness [85].

##### Nanoparticles and 18F-FDG in Theranostic Applications for Colon Cancer

In colon cancer, combining 18F-FDG with nanoparticles allows for dual-purpose applications: precision imaging and therefore targeted therapy. Such a theranostic strategy allows for PET imaging of tumor growth at the same time as targeted drug delivery to the tumor site. For example, multifunctional nanoparticles can encapsulate 18F-FDG and chemotherapy drugs such as 5-fluorouracil (5-FU), which work as both imaging and therapeutic agents. This enables the clinicians to closely track the response to treatment with the help of PET scans of tumor activity and metabolic changes Nanoparticles may also be conjugated with both 18F-FDG and radiosensitizers to enhance the susceptibility of cancer cells to radiation during radiotherapy [86].

#### 3.1.2. 99mTc-Labeled Agents: 99mTc-HYNIC-D(TPPE)

Technetium-99m (99mTc) is widely utilized in nuclear medicine due to its beneficial properties. It has a relatively short half-life of approximately 6 h and releases gamma rays at 140 keV, making it ideal for imaging purposes. By attaching targeting molecules, 99mTc can selectively bind to specific tissues or cell receptors, allowing for its application in diagnostic imaging [86,87].

99mTc does not specifically focus on cancer or any other medical condition. In order to be effective for diagnostic purposes, 99mTc is chemically attached to targeting molecules such as antibodies, peptides, or small molecules. These targeting agents are created to attach to particular biological markers found on or inside cells (such as receptors, antigens, or enzymes) that are frequently found to be overexpressed in tumors or inflamed tissues [86,87].

##### 99mTc-HYNIC-D(TPPE)

99mTc-HYNIC-D(TPPE) is a promising imaging probe that demonstrates great potential in detecting colon cancer. This radiopharmaceutical utilizes Technetium-99m (99mTc) as its radionuclide, which is attached to a specific ligand system for targeting cancer cells. HYNIC, a bifunctional chelator, binds the radionuclide to the tumor-targeting molecule D(TPPE), which is a derivative of TPPE (Triphenylphosphonium). The TPPE moiety has a remarkable ability to target tumors by easily penetrating mitochondrial membranes. This is made possible by the high mitochondrial membrane potential found in tumor cells [88,89].

##### Mechanisms and Considerations for 99mTc-HYNIC-D(TPPE) as a Radiopharmaceutical Agent in Colon Cancer Imaging

Targeting Tumors: TPPE, the active targeting component, is able to accumulate in tumor cells by taking advantage of the higher mitochondrial membrane potential found in these cells as compared to normal cells. It is commonly observed that tumor cells have a different bioenergetic state compared to normal cells. This altered state results in a more negative mitochondrial membrane potential, which has the stimulating effect of attracting molecules based on TPPE [88,89].

Cellular Uptake and Accumulation in Tumor Cells: After administration, 99mTc-HYNIC-D(TPPE) travels through the bloodstream and selectively gathers in cancerous tissues. This is because TPPE has a strong attraction to the mitochondrial membranes found in tumor cells. TPPE, due to its lipophilic cation nature, has the ability to traverse both plasma and mitochondrial membranes. This movement is facilitated by the negative charge present within the mitochondria of cancer cells. This mechanism results in the targeted accumulation of the imaging agent in colon cancer cells [88,90].

Potential in Colon Cancer Imaging: The unique property of TPPE to target mitochondria makes 99mTc-HYNIC-D(TPPE) a valuable tool for imaging colon cancer cells. These cells often have abnormal bioenergetic states and increased mitochondrial activity. By focusing on specific areas, we can enhance the ability to detect cancer at an early stage, pinpoint the exact location of tumors, and assess the presence of cancer spread to the liver or other parts of the body [88,90].

Diagnostic Imaging and SPECT Utility: When it comes to cancerous tissues, the visualization of 99mTc-HYNIC-D(TPPE) can be achieved through Single-Photon Emission Computed Tomography (SPECT). This imaging technique enables a comprehensive view of the tumor site, offering crucial diagnostic insights into the size, location, and activity of the tumor. Colon cancer can be effectively evaluated for metastatic spread using SPECT, which is a valuable tool in scientific research [88,91].

### 3.2. Nano-Targeted Radiopharmaceuticals for Colon Cancer Therapy

**Figure 3:**
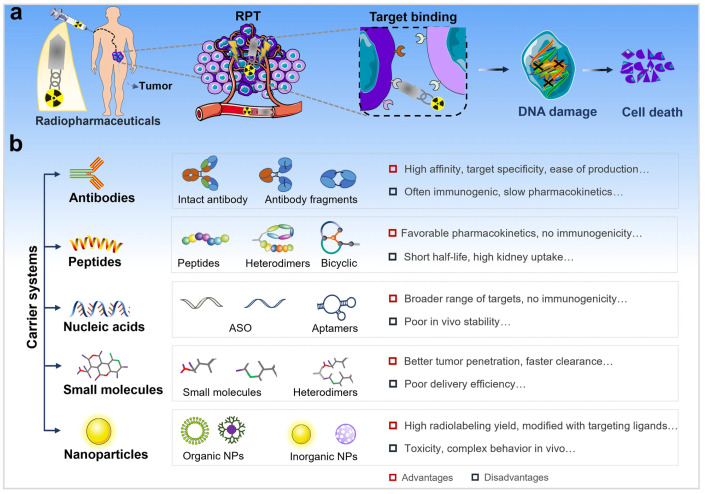
(**a**). An For targeted therapy, carriers of radiopharmaceuticals that have a high affinity and specificity for the tumor target can direct therapeutic radionuclide payloads to tumor cells in a selective manner. (**b**). The advantages and disadvantages of different carriers, as well as the primary types of common carrier systems utilized for radiopharmaceutical research [94].

Radiopharmaceutical therapy (RPT) is emerging as a promising cancer treatment that involves the targeted delivery of radiation (Figure 3). In RPT, radiation is administered systemically or locally using pharmaceuticals that either preferably bind to cancer cells or accumulate through physiological processes. Importantly, the radionuclides employed in RPT also radiate photons that can be imaged, allowing for non-invasive surveillance of the therapeutic agent’s biodistribution. RPT has demonstrated efficacy when compared to other systemic cancer treatment options, while expressing minimal toxicity [92,93].

#### 3.2.1. Neurotensin (NT) Radiolabeled with 68Ga and 177Lu

In recent years, there has been increasing interest in developing drugs that can serve a dual purpose, treating the malignant tissue through irradiation of localized areas as well as imaging specific targets like tumors, while minimizing harm to the rest of the body. The growing interest in theranostic applications has driven the development of peptides that can be coupled with pairs of imaging and therapeutic radioisotopes. These peptides have the potential to quickly localize to cancer cells, which allows them to deliver the therapeutic or diagnostic effect directly to the tumor site (Table 3) [66,94].

**Figure 4:**
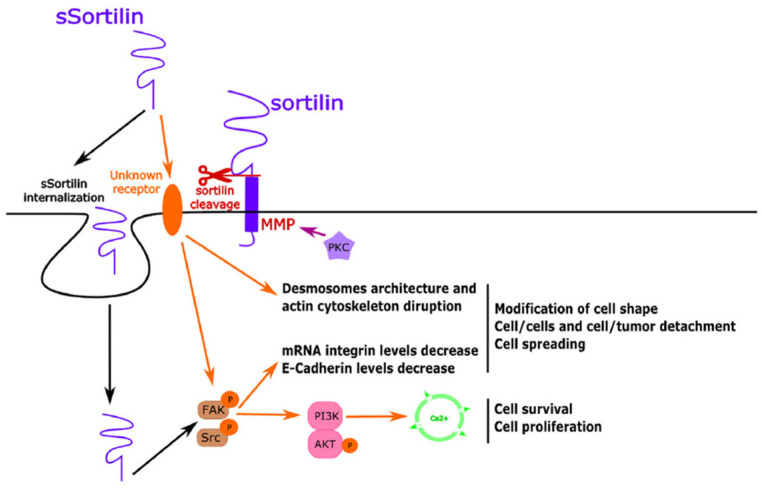
(sSortilin) in colorectal cancer. The sSortilin/unknown receptor complex disrupts the Desmosome architecture, disorganizing actin filaments. This leads to increased integrin mRNA levels, decreased E-Cadherin protein expression, and metastasis formation. The sSortilin/unknown receptor complex activates FAK/Src pathways, which can also stimulate PI3K/AKT activation and Ca^2+^ release, promoting cell survival and cell proliferation [100].

One example of a molecule with theranostic potential is the neurotensin (NT) peptide. Neurotensin (NTS) is a naturally occurring hormone that influences the function of the gastrointestinal (GI) tract. Earlier studies have demonstrated that NT has the ability to target and localize to various types of tumors, including pancreatic cancer, colorectal cancer, lung cancer, prostate cancer, and breast cancer. NTS acts through its cellular receptors (NTSRs), which have been associated with the carcinogenesis of several cancers. In colorectal cancer (CRC), there is considerable evidence from both in vitro and in vivo studies that explicate the molecular biology of NTS/NTSR signaling and its effect on the growth of CRC cells. Moreover, modifying and blocking the NTS/NTSR signaling route seems to decrease CRC growth in both animal and cell culture studies (Figure 4) [95,98,99].

The outcome of NT is mediated through mitogen-activated protein kinases, epidermal growth factor receptors, and phosphatidylinositol-3 kinases. Their involvement in promoting tumor cell proliferation, migration, and DNA synthesis has been investigated across various types of cancer. Evers et al. exhibited the presence of NT/N gene expression, NT peptide content, and functional NTR in various human colon cancers [101,102].

The potential for detecting colon cancer using the NT (neurotensin) peptide coupled with the radioactive isotope 68Ga is indicated by the results reported. Additionally, the ability to couple the NT peptide with 177Lu suggests that it could have therapeutic applications as a theranostic agent, both with and without prior inhibition of peptidases. In vitro studies have demonstrated increased uptake and retention of the NT peptide in HT-29 colon cancer cells. Furthermore, in vivo biodistribution data have shown that the NT peptide can effectively target tumor sites. However, further studies are still needed to assess whether long-term accumulation of 177Lu-DOTA-NT in organs like the kidneys, liver, or bone marrow could lead to toxic effects [95].

#### 3.2.2. Precision Treatment of Colorectal Cancer Through Radiolabeled Antibodies

Antibodies tagged with a radioactive isotope are known as radiolabeled antibodies. These antibodies can be conjugated with radioactive isotopes for therapeutic and diagnostic applications. The antigens on the membranes of immune cells or cancer cells in the tumor microenvironment are the target of these antibodies [103,104].

**Figure 5:**
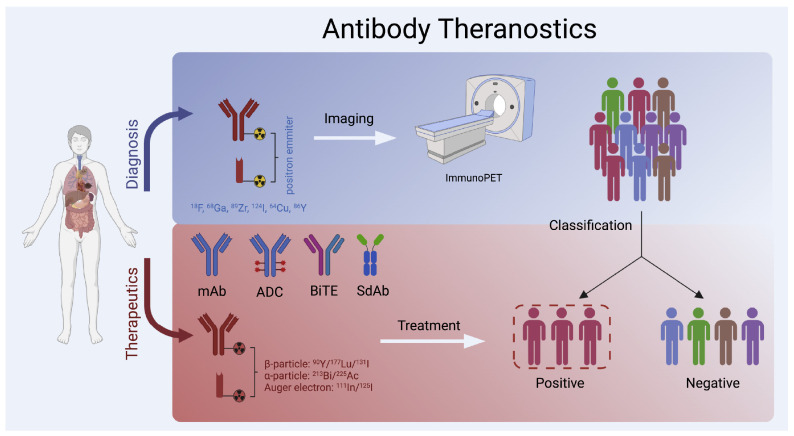
Using antibody theranostics in clinical settings for precision medicine. After labeling antibodies or antibody fragments with the proper radionuclides for non-invasive molecular imaging of significant targets and patient selection, non-radioactive antibody therapies or therapeutic antibody radiopharmaceuticals are administered for treatment [104].

**Figure 6:**
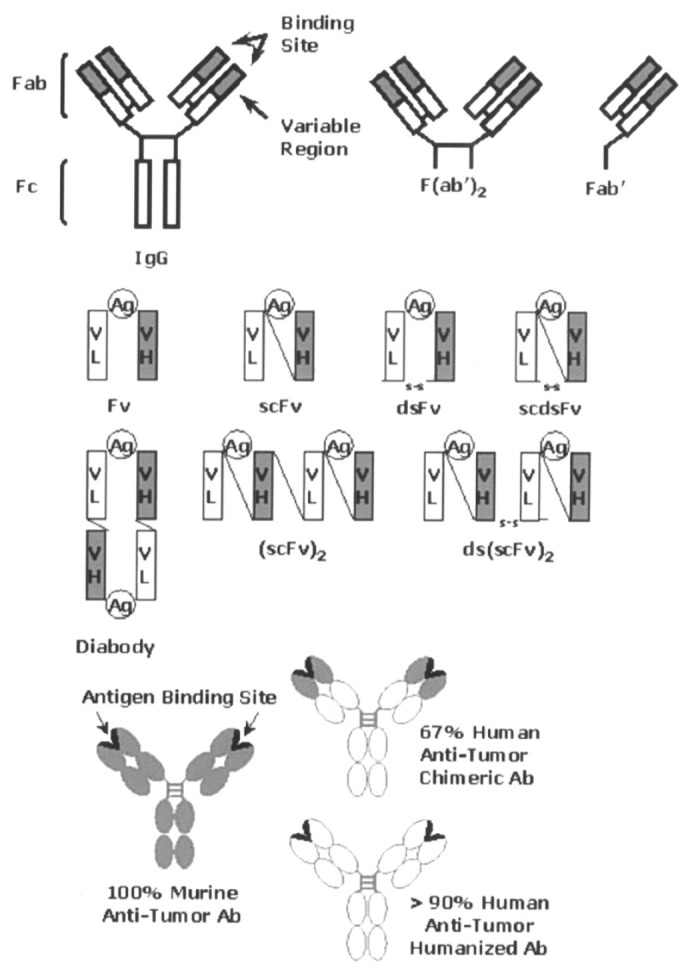
Diagram showing the various antibody-derived targeting molecules under investigation in RAIT, such as diabody, chimeric, intact IgG, F(ab′)2, Fab′, single-chain Fv, and CDR-grafted (humanized) antibody constructions [105].

Monoclonal antibodies (mAbs) can potentially identify and target tumor cell antigens. This exclusive feature has led to their application in delivering radioisotopes to tumor sites for scintigraphy imaging and radioimmunotherapy (RIT) (Figure 5). The key role of carrier molecules in radiopharmaceutical applications has been observed, while emphasizing the use of monoclonal antibodies (mAbs) as targeted delivery vehicles for cancer therapy. The choice of carrier is crucial for the successful application of radiopharmaceuticals (Figure 6) [104].

Studies have recently been conducted on the use of mAbs and their derivatives as carrier molecules, highlighting the novel strategies using radiolabeled mAbs/derivatives for lymphoma and colorectal cancer treatment. These radiolabeled constructs are being employed for targeted cancer therapy, particularly in lymphoma and colorectal cancer. The use of radiolabeled antibodies focusing on key colorectal cancer antigens has been extensively investigated as a therapeutic modality for the treatment of colorectal cancer (CRC) patients. The most frequently targeted antigens for these radiolabeled antibody therapies include carcinoembryonic antigens (CEAs), epithelial cell adhesion molecules, and the colon-specific antigen p. Targeting these tumor-associated antigens with radiolabeled antibodies represents a promising strategy for the selective delivery of therapeutic radiation to CRC tumors. Identifying the appropriate antigens for cancer cells can be difficult, and so far, monoclonal antibodies (mAbs) have proven more efficacious against certain cancers than others [106,107].

#### 3.2.3. Imaging Modality Used for Radiolabeled Antibodies for Precise Treatment of Colorectal Cancer

Radiolabeled antibodies for precise treatment of colorectal cancer summarized in (Table 4).

#### 3.2.4. Chemo-Radiotherapy with 177Lu-PLGA(RGF)-CXCR4L for the Targeted Treatment of Colorectal Cancer

Regorafenib (RGF) is the first authorized small-molecule multi-kinase inhibitor employed for treating patients with metastatic colorectal cancer (MCC) and metastatic gastrointestinal stromal tumors (GISTs) that have not responded to conventional chemo- and immunotherapy. REG blocks the activity of protein kinases involved in the regulation of tumor angiogenesis, oncogenesis, and the tumor microenvironment [111].

Nanoparticles have been utilized in various applications, including cancer treatment, targeted drug delivery systems, and clinical bioanalytical diagnostics. C-X-C Chemokine receptor type 4 (CXCR4) is the most frequently expressed chemokine receptor across more than 23 types of human cancers, including colorectal cancer. Rhodopsin-like G protein-coupled CXCR4 is the GPCR with dominant expression. In colorectal cancer, the CXCR4/CXCL12 axis plays a role in tumor development, invasion, angiogenesis, and metastasis [112].

One study aimed to synthesize and preclinically evaluate a targeted nanosystem for chemo-radiotherapy in colorectal cancer, using RGF encapsulated in Poly(D,L-lactic-co-glycolic acid) (PLGA) nanoparticles coated with a CXCR4 ligand (CXCR4L) and 177Lu as a therapeutic β-emitter. 177Lu-PLGA(RGF)-CXCR4L nanoparticles decreased cell viability and proliferation by inhibiting Erk and Akt phosphorylation and promoting apoptosis. Moreover, in vivo administration of 177Lu-PLGA(RGF)-CXCR4L significantly reduced tumor growth in an HCT116 colorectal cancer xenograft model. The biokinetic profile showed hepatic and renal elimination. In this research, a new nanoparticle system based on Regorafenib (RGF) was designed and synthesized as a chemotherapeutic agent entrapped in PLGA nanoparticles. In addition, 177Lu was used as the radiotherapeutic component to produce a synergistic anticancer effect specifically targeted to cancer cells through the CXCR4 ligand [113].

#### 3.2.5. Other Available Techniques for Colon Cancer Treatment

- **Surgery:** The main therapy for resectable CRC is surgical excision. Surgery is the preferred therapy option for patients with early-stage colorectal cancer (CRC), where healing is attained by excising the tumor along with a portion of healthy intestine. In inoperable CRC, standard therapies include chemotherapy, radiotherapy, and immunotherapy. However, these therapies have certain limitations, including their non-specific and cytotoxic effects on normal healthy cells, which result in secondary complications [114,115].
- **Chemotherapy:** Cytotoxic drugs approved for CRC help to slow disease development and extend individual lifespan. The approved medications include irinotecan, fluoropyrimidines, oxaliplatin, trifluridine-tipiracil, 5-fluorouracil (5-FU), and capecitabine, which are primarily used as chemotherapeutic agents for curing CRC. Fluorouracil (5-Fu) and oxaliplatin are the primary treatment agents utilized for CRC chemotherapy, and 5-Fu-based chemotherapy regimens are commonly used in CRC patients.

However, drug resistance often leads to incurable recurrent CRC, necessitating the development of novel approaches with significant benefits and minimal drawbacks. Chemotherapy agents have several limitations, including systemic toxicity, poor tumor-specific selectivity, and massive damage to normal cells while killing tumor cells, prompting the exploration of molecular targeted therapies [114,116,117].

- **Radiotherapy:** Radiotherapy is also a promising option for CRC patients. However, it has some plausible and long-term toxicity effects on vital organs that must be overcome by modifying radiation intensities. Different specialized radiotherapy techniques for colorectal cancer have been suggested for varying tumor stages. While radiotherapy provides effective control locally, concerns persist regarding treatment side effects, local recurrence, and distant metastasis.

The current standard for radiotherapy treatment is three-dimensional conformal radiotherapy (3DCRT), which enables target localization and dose assessment for the target volume and organs at risk (OARs) through 3D planning and dose volume histograms. Three-dimensional conformal radiotherapy (3D-CRT) was previously the preferred method of treatment. It employs CT scanning to reconstruct the tumor volume and specifically direct beams to target it, creating a central cuboid area of intense irradiation. However, despite its precision, it cannot completely avoid exposure to the surrounding healthy tissues [118,119].

- **Nano-radiopharmaceuticals** represent a promising approach to treating colon cancer, combining nanotechnology and nuclear medicine to deliver radiation therapy selectively to specific organs or tissues. These nanoparticles target specific cancer cells, reducing harm to healthy cells and tissues. They work by integrating radiopharmaceuticals inside their structure, which are then transported to tumor sites in the patient’s body. The benefits of nano-radiopharmaceuticals include targeted therapy, enhanced imaging, improved drug delivery, personalized medicine, and reduced toxicity. Current research focuses on developing new nanomaterials and nanosystems for radiopharmaceutical delivery, investigating different radionuclides and their combinations, and designing targeted nano-radiopharmaceuticals that selectively accumulate in cancer cells. Future prospects include targeted therapy, personalized medicine, and combination therapies. Overall, nano-radiopharmaceuticals offer a promising approach to colon cancer treatment, improving patient outcomes and quality of life [3,12].

### 3.3. Using of Nanoparticles or Surface-Functionalized Nanoparticles in Both Diagnostic and Therapeutic Purposes

Radiodiagnostic and radiopharmaceutical nanoparticle therapeutics with surface functionalities are used for diagnostics and the treatment of colon cancer. Such is the huge promise that surface-functionalized NPs hold for application as radiopharmaceutical agents in diagnostics and the treatment of colon cancer. These nanoparticles of material compositions such as gold, silica, or lipids are usually functionalized by the attachment of appropriate functional groups, ligands, or antibodies targeting the specific biomarkers on the colon cancer cells. This makes both the precision of imaging and therapeutic interventions enhanced with minimal systemic side effects.

#### 3.3.1. Diagnostic Applications

The sensitivity and specificity of the imaging modalities such as PET and SPECT can be improved by surface-functionalized nanoparticles. Conjugation of radiolabels such as Tc-99m or F-18 to nanoparticles may significantly improve the targeted imaging of colon cancer lesions. The functionalization with tumor-specific ligands, such as folic acid or antibodies against CEA, will ensure in advance that only malignant tissues accumulate selectively for superior contrast and reduced false positives [120].

#### 3.3.2. Therapeutic Uses

Surface-functionalized radiopharmaceutical nanoparticles are therapeutically able to provide targeted radionuclide delivery to cancer cells while minimizing damage to healthy tissues. Alpha-emitting isotopes, such as Actinium-225 (Ac-225), or beta-emitting isotopes, such as Lutetium-177 (Lu-177), can be incorporated into nanoparticles for localized radiation therapy. Functionalization with polyethylene glycol (PEG) or tumor-penetrating peptides further enhances bioavailability and tumor retention. These recent developments provide a new horizon for personalized treatment approaches, especially in patients suffering from metastatic or chemoresistant colon cancer.

#### 3.3.3. Combination Therapies

Such versatility in surface-functionalized nanoparticles can facilitate their integration into different modalities. The capability of nanoparticles that are functionalized with both imaging agents and therapeutic radionuclides to enable theranostic applications provides the possibility of monitoring therapeutic efficacy in real time. These platforms can be coengineered to deliver chemotherapeutic agents for amplifying therapeutic impact through synergistic effects [121,122,123].

## 4. The Development of Colon Cancer Drugs with Radionuclides and Nanostructures

Colon and colorectal cancers are one of the most common types of malignancies worldwide and greatly contribute to morbidity and mortality from cancer [124]. These are cancers of the epithelial cells lining the colon or rectum and most of the time start as benign polyps that take many years to turn malignant. Risk factors include age; genetic predisposition—possibly from Lynch syndrome or familial adenomatous polyposis; life habit factors like the consumption of a diet with high amounts of red and processed meats; obesity; smoking; and a sedentary lifestyle. It should be expected that in most early stages of CRC, it presents asymptomatic symptoms, but can be symptomatic where an advanced form of the disease has taken over through rectal bleeding, changing bowel habits, abdominal pain, and unexplained weight loss, among other symptoms. Improvements in different screening modalities, such as colonoscopy and fecal immunochemical testing, have facilitated early detection and improved survival. Newer treatments, including immunotherapy and targeted therapies, hold promise for personalized interventions, especially in metastatic or recurrent disease [125].

This shows that nanotechnology can bridge biological and physical sciences by means of nanostructures or nanophases across various fields of science [126], especially in nanomedicines and nano-based drug delivery systems, wherein such particles hold major interest [127]. In the nanotechnology era, drug delivery in oncology has become more advanced. In fact, nanostructures combined with appropriate radionuclides open unparalleled opportunities for the specificity, efficacy, and safety of therapies against colon cancer. This discussion summarizes some of the developments in the field, discussing the role of radionuclides, versatile nanostructures, and the promise of theranostic approaches [128].

### 4.1. Radionuclides in the Therapy of Colon Cancer

Radionuclides have been one of the cornerstones in nuclear medicine because they can emit radiation that visualizes or destroys cancer cells. Beta emitters, such as Yttrium-90 and Lutetium-177, are particularly suited for treating solid tumors due to their tissue-penetrating properties. Alpha emitters, such as Actinium-225, have high LET and therefore lead to localized and potent cytotoxic effects Radionuclide therapies are effective for the palliation of advanced colon cancer, but systemic toxicity has remained a limiting factor [129,130].

### 4.2. Nanostructures as Multifunctional Platforms

Nanostructures include liposomes, polymeric nanoparticles, gold nanoparticles, and mesoporous silica nanoparticles; all these methods are customizable options for the efficient delivery of radionuclides into cancerous foci. That is, functionalization with certain tumor-targeted ligands, such as monoclonal antibodies, peptides, and aptamers, enables them to be precisely delivered into tumor cells while normal tissues are spared. One example is how mesoporous silica nanoparticles coloaded with Yttrium-90 have actually demonstrated efficient tumor uptake in addition to fewer off-target effects in models [131]. In addition, liposomal nanocarriers of Lutetium-177 have also exhibited higher therapeutic efficacy owing to increased tumor penetration from the EPR effect. Gold nanoparticles have recently emerged as promising vehicles for delivering radionuclides due to their excellent biocompatibility and ease of performing surface modifications, with the possibility for imaging and therapy [132,133].

### 4.3. Theranostic Nanostructures: Where Diagnostics Meet Therapy

The concept of theranostics—combining diagnostic imaging and therapy within a single nanostructure—has gained great momentum in colon cancer management. For instance, dual-labeled nanoparticles, incorporating Gallium-68 for PET imaging and Lutetium-177 for therapy, provide the perfect tumor localization and treatment [134]. Such functionality has been implemented with Actinium-225-functionalized quantum dots during their preclinical examination for metastatic colon cancer, demonstrating real-time imaging and an on-spot alpha radiation of the lesions [135,136].

### 4.4. Stability and Safety: Challenges Overcome

However promising, there are several challenges to radionuclide-based nanostructures. It is very critical for radiolabeling to be stable, since this would prevent its early release; if there is an early release of the radionuclide, it will enhance systemic toxicity. Improvements in chelator chemistry, e.g., the synthesis of DOTA and NOTA derivatives, have achieved a great deal for the stability of radionuclides with improved properties [137]. Second, nanoparticles have to be carefully assessed for their safety profile, as prolonged circulation time may be translated into potential accumulation within nontarget organs, such as the liver and spleen. To that end, different approaches using biodegradable substrates and stealth coatings—for example, polyethylene glycol—have been developed to address these issues [138].

### 4.5. Future Directions and Clinical Translation

Therefore, interdisciplinary collaboration will further accelerate the clinical translation of these technologies. Artificial intelligence and computational modeling can be combined to design nanoparticles with optimized physicochemical properties for enhanced tumor targeting [139]. A combination of nanostructures loaded with radionuclides and immune checkpoint inhibitors has also been explored and is considered to represent one of the most promising approaches in overcoming treatment resistance in advanced disease More recently, advances in microfluidics have enabled the scalable and reproducible synthesis of nanoparticles, one of the major hurdles in clinical translation [140]. Clinical trials currently underway will yield important information regarding safety and efficacy in patients with colon cancer treated by radionuclide–nanostructure therapies.

The integration of radionuclides with nanostructures opens up new dimensions in the field of colon cancer treatment. Combining all these factors—spectral tumor targeting, reduced off-target toxicity, and theranostic capabilities—into novel innovations may redefine the standard treatment paradigm. Continued research and development will be very important, coupled with regulatory and manufacturing development, to ensure clinical translation for this promising therapeutic strategy.

## 5. Challenges and Limitations of Nano-Radiopharmaceuticals in Colon Cancer

Colon cancer treatment using nano-radiopharmaceuticals poses various challenges and limitations. A significant issue to consider is the unintentional accumulation of nanoparticles in healthy tissues, leading to potential long-term toxic side effects. This matter holds great importance due to the potential long-term toxicity that can arise from the continuous or repeated administration of these nanoparticles, which can adversely affect vital organs like the liver, kidneys, and spleen. In addition, there is a significant hurdle to overcome in terms of the expensive production costs. This encompasses the intricate procedures required for nanoparticle synthesis, radiolabeling, and quality control. These factors contribute to the overall cost, making it challenging to implement nano-radiopharmaceuticals on a large scale in clinical settings. In addition, the process of gaining approval and implementing these treatments is made more complex by regulatory obstacles. Extensive testing and validation are necessary to ensure their safety and effectiveness [141].

### 5.1. Challenges and Prospects of Combining Nanoparticles with 18F-FDG for Colon Cancer Treatment

Despite having identified the synergistic opportunities of using nanoparticles and 18F-FDG in theranostics in the treatment of colon cancer, some challenges need to be met. One big issue surrounds the biocompatibility and safety of the nanoparticles; some materials could trigger an immune response, and others can be toxic. However, in the manufacture of nanoparticle-based systems, maintaining control over the structure and function is challenging and presents certain legal barriers that need to be addressed before these systems can be applied in clinical practice. Recent work primarily presents the creation of new nanomaterial constructs to maximize drug relaxation and picture definition and reduce toxicity [89].

In summary, the use of nanoparticles incorporated with 18F-FDG in colon cancer theranostics shows a promising future for better diagnosis and treatment of the cancer. It is stated that organizing both imaging and therapy into one linked solution is beneficial, since it offers therapy as exactly as possible for the patient. Despite these obstacles, a great deal of continuous improvement in nanomedicine related to colon cancer theranostics is anticipated in the future.

### 5.2. Toxicity Concerns

The biocompatibility and safety of nano-radiopharmaceuticals are crucial considerations that have a direct impact on their therapeutic efficacy and potential hazards. When nanoparticles (NPs) are utilized in patient treatment, there is the potential for unintended consequences, where they might gather in unaffected tissues and organs. Observations from preclinical studies have revealed an unintended deposition, which has sparked concerns regarding potential toxicity and long-term health implications. The accumulation of NPs in these organs may hinder their performance or result in negative consequences, especially when administered repeatedly. Thus, it is crucial to develop nanoparticles using materials that can easily degrade or are highly compatible with living organisms. The unique characteristics of these nanoparticles enable them to be safely broken down or processed by the body, without any negative effects. However, they still retain their remarkable capability to deliver targeted radiation therapy to cancerous cells with great efficiency. Finding the right balance between reducing toxicity and maintaining therapeutic effectiveness is a significant hurdle in the development of nano-radiopharmaceuticals for medical applications [142].

Targeted radionuclide therapy has proven to be a powerful method in cancer treatment; even so, some patients may not show the expected results from the selected protocol, while others may worsen or develop resistance to the treatment protocol, as reported by Rinne and colleagues. As a consequence, combining two radionuclides might be a favorable approach [143,144].

### 5.3. Manufacturing and Cost

Nano-radiopharmaceuticals are synthesized in a relatively tedious and cost-intensive manner through production processes such as nanoparticle synthesis, radionuclide incorporation, and quality assurance processes. These processes may be expensive and, therefore, may not ensure the widespread availability of nano-radiopharmaceuticals to other healthcare facilities, with treatments being limited to only a few patients. However, additional issues with regulation concerning the approval and commercialization of these agents pose further impediments to their use in clinics [111,145].

Nano-radiopharmaceuticals are an improvement over previous techniques of colon cancer imaging and treatment since they enable better targeting of tumor cells, provide better diagnostic resolution, and provide therapies. Despite the fair amount of promise they display, especially in preclinical and early-phase clinical trials, these technologies, to become part of the standard of care in clinical medicine, must overcome difficulties concerning safety, toxicity, the cost of manufacturing, and drug approval. To overcome these drawbacks and extend the opportunities offered by nano-radiopharmaceuticals for the further enhancement of the prognosis of colon cancer, further research and development are required [112].

This section has discussed the current state of nano-radiopharmaceuticals in relation to colon cancer, focusing on their diagnostic and therapeutic uses, as well as the challenges that need to be addressed in order to fully realize the potential of these advancements. Nano-radiopharmaceuticals applied to colon cancer will only increase in the future, as recent studies are still promising new advancements that will shift the landscape of cancer treatment [94].

### 5.4. Biodistribution and Toxicity

A major challenge in the clinical application of nano-radiopharmaceuticals is the issue of biodistribution and toxicity. Nanoparticles can accumulate in non-target tissues due to their small size and surface properties, resulting in off-target effects and toxicity. For example, the reticuloendothelial system (RES) captures these nanoparticles, leading to their accumulation in organs such as the liver and spleen, which can be toxic [146].

### 5.5. Limited Clinical Data

In spite of the promising results of preclinical studies on nano-radiopharmaceuticals, clinical data supporting their efficacy and safety in humans are lacking. There are many differences in metabolism, immune response, and tumor biology between animal models and humans, which can affect the performance of these nanomaterials [147].

### 5.6. Complexity of Tumor Microenvironment

Another limitation is the heterogeneity of the tumor microenvironment. There are several factors that can impede the effective delivery of nano-radiopharmaceuticals to the tumor site, such as abnormal vasculature, hypoxia, and high interstitial fluid pressure. As a result of these barriers, nanoparticles are unable to achieve the therapeutic efficacy they are meant for, and their behavior in different types of tumors cannot be predicted [148].

## 6. Future Direction and Development

### 6.1. Development of More Targeted Radiopharmaceuticals

Advancing the progress in the development of more precise radiopharmaceuticals is crucial for enhancing the diagnosis and treatment of colon cancer. There is ongoing research dedicated to improving the specificity and effectiveness of agents used for colon cancer, such as ^18^F-fluorodeoxyglucose (^18^F-FDG) and 99mTc-labeled agents [65,149].

### 6.2. Future Studies for ^18^F-Fluorodeoxyglucose (^18^F-FDG) as a Radiopharmaceutical

- **Enhancing specificity:** Although ^18^F-FDG is drawn to regions with elevated glucose metabolism, it is not exclusive to cancer cells, which can result in misleading results such as inflammation being mistaken for cancer. Further research could explore new glucose analogs or other metabolic tracers that specifically target the unique metabolic pathways of colon cancer [150,151].
- **Combination imaging:** The integration of different tracers or modalities like MRI or CT with ^18^F-FDG has the potential to enhance diagnostic accuracy, enabling a clearer differentiation between cancerous and non-cancerous lesions [149,150,151,152].

### 6.3. Future Studies for 99mTc-Labeled Agents: 99mTc-HYNIC-D(TPPE) as a Radiopharmaceutical

- **Tumor-targeted delivery:** Future research may involve the modification of compounds labeled with 99mTc, such as 99mTc-HYNIC-D(TPPE), to enhance their ability to specifically target receptors or biomarkers associated with colon cancer. Utilizing peptides or antibodies conjugated to 99mTc has the potential to greatly enhance the specificity of tumor targeting, helping to distinguish between cancerous and non-cancerous lesions [65,149,150,151,152].
- **Nanoparticle conjugation:** The incorporation of 99mTc into nanoparticles shows promise in enhancing the targeted delivery to colon cancer cells. Studies have demonstrated that nanoparticles can boost the concentration of tracers at tumor sites by taking advantage of the enhanced permeability and retention (EPR) effect. This finding opens up new possibilities for enhancing accumulation in tumors through scientific research [149,150,151,152,153,154,155].

### 6.4. Future Perspectives

177Lu-PLGA(RGF)-CXCR4L requires additional preclinical safety trials and clinical evaluation as a potential combined treatment for colorectal cancer. Also, identifying patients with colon cancer who respond to NT could have significant future implications for developing therapeutic agents that inhibit the effects of NT.

Treatment through radiolabeled monoclonal antibodies requires further improvements in radiochemistry, and the scale of production may help to reduce the high production costs and allow for the wider use of these therapies. The opportunities for RIT are increasing, and further trials will demonstrate the potential of this approach in treating cancer patients. Moreover, in translational trials for CRC therapy, antagonists of the CXCR4/CXCR7/CXCL12 axis need to be investigated in combination with chemotherapy, angiogenesis inhibitors, or immunotherapy.

## 7. Conclusions

Colon cancer is one of the most common cancer-related deaths worldwide; the diagnosis and treatment of the disease present many difficulties. Conventional diagnostic approaches are comparatively less accurate for defining the extent of tumor growth or metastasis, and commonly applied treatments involve undesirable side reactions due to their imprecise nature. The development of nano-radiopharmaceuticals has brought a new concept in cancer treatment, with a view to advanced imaging and therapeutic procedures. They are innovative agents that integrate the features of nanotechnology with the diagnostic and therapeutic potential of radionuclides, allowing for targeting tumor localization and the delivery of the therapeutic radiation dose to cancer cells. Recent progress made in this area has demonstrated high potential. Currently, nano-radiopharmaceuticals are studied mainly in the context of colon cancer using nano-devices, both for imaging and treatment purposes.

In conclusion, the use of nano-radiopharmaceuticals in the treatment of colon cancer is a promising area of research. The findings of this review highlight the potential of nano-radiopharmaceuticals to enhance the efficacy of external radiation therapy and targeted drug delivery. However, further studies are needed to fully explore the potential of nano-radiopharmaceuticals in the treatment of colon cancer, for example, studies on the safety and long-term effects of using 177Lu-PLGA(RGF)-CXCR4L in clinical settings. Future research should focus on developing new and more effective nano-radiopharmaceuticals, like highly advanced radiopharmaceuticals for targeting CRC biomarkers rather than those based on high glucose levels, as well as investigating their safety and efficacy in clinical trials. Also, more clinical studies are needed on the role of targeted radionuclide therapy related to developed drug resistance. Overall, the use of nano-radiopharmaceuticals in the treatment of colon cancer has the potential to improve treatment outcomes and quality of life for patients.

## Figures and Tables

**Table 1:** Advantages and disadvantages of nanoparticles as drug delivery systems [13].

Nanoparticles Type	Advantages	Disadvantages
Liposome	Liposomes exhibit better properties, including site-targeting, sustained or controlled release, protection of drugs from degradation and clearance, superior therapeutic effects, and lower toxic side effects.	Liposomes can be sensitive to environmental conditions, leading to instability, synthesis of liposomes can be complex, materials and processes involved in producing high-quality liposomes can be expensive, poor stability, could crystallize after prolonged storage conditions.
Quantum Dot	Ideal for long-term applications in imaging and sensing, can be made from various semiconductor materials for diverse applications, biocompatible.	Many contain heavy metals, synthesis of high-quality quantum dots can be complex and expensive, may degrade or release toxic components in biological settings.
Magnetic nanoparticles	Targeted drug delivery, used in imaging, drug delivery, and remediation, increased loading capacity for drugs, biocompatible.	Potential toxicity from heavy metals or degradation, can agglomerate or degrade over time, production can be complex and costly, precision targeting can be challenging.
Dendrimer	Precise, repetitive branching allows for uniformity, increased functional groups enhance drug loading and reactivity, can be easily modified for various applications, targeted delivery	Complex synthesis, synthesis and purification can be expensive, may degrade under certain conditions, affecting performance, low loading capacity
Gold nanoparticle	Generally non-toxic and well tolerated in biological systems, strong optical properties, efficiently encapsulates and delivers therapeutic agents, used in photothermal therapy to destroy cancer cells.	Synthesis and purification can be expensive, may aggregate over time, affecting performance, potential toxicity
Carbon Nanotube	Water-soluble, less toxic, provide a large surface area for enhanced interactions, excellent conductivity, useful in electronic and photonic applications, high thermal stability allows for use in extreme conditions.	Can clump together, reducing effectiveness in applications, complex synthesis, limited functionalization
Micelle	Enhanced drug solubilization, targeted delivery, capable of controlled and sustained release of therapeutics, improve the solubility of hydrophobic drugs	Stability issues, limited drug loading capacity, size may restrict penetration into certain tissues, complex formulation
Mesoporous Nanoparticle	High drug and gene loading capacity, tuneable pore size, large surface area, biocompatible and biodegradable, controlled porosity	Expensive, not enough information about cytotoxicity, biodistribution, biocompatibility, low stability, formation of aggregates, hemolysis

**Table 2:** The half-life of the most common radionuclides used in nano-radiopharmaceuticals for colon cancer diagnosis and treatment [65,66,67].

Type	Radionuclide Used in Nano-Radiopharmaceuticals	Half-Life	Reference
Diagnostic Radionuclides	Technetium-99m (Tc-99m) Iodine-123 (123I) Fluorine-18 (F-18)	Half-life of 6 h Half-life of 13.2 h Half-life of 109.7 min	[65]
Therapeutic Radionuclides	Yttrium-90 (Y-90) Actinium-225 (225 Ac) Lutetium-177 (Lu-177)	Half-life of 64.1 h Half-life of 10 days Half-life of 6.6 days	[68]
Diagnostic And Therapeutic Radionuclides	Copper-64 (Cu-64) Zirconium-89 (Zr-89)	Half-life of 12.7 h Half-life of 79 h	[67]

**Table 3:** Summary of the imaging modality used: neurotensin (NT) radiolabeled for colon cancer indications [95,96,97].

Aspect	Description	References
68Ga-NT in PET Imaging for Colon Cancer	In order to radiolabel NT with 68Ga, the radionuclide is bonded to NT using a chelator such as DOTA (1,4,7,10-tetraazacyclododecane-N,N′,N″,N′′′-tetraacetic acid). The 68Ga-DOTA-NT compound is subsequently synthesized for therapeutic use.	[95]
Imaging Application	Using PET imaging with 68Ga-NT, it is possible to visualize lesions in the colon that express neurotensin receptors, providing valuable insights into colon cancer. Through meticulous localization and evaluation, the technology enables the identification of primary tumors, metastases, and any remaining disease, leading to improved detection.	[96,97]

**Table 4:** Radiolabeled antibodies for precise treatment of colorectal cancer [108,109,110].

Aspect	Description	Advantages	Disadvantages	Reference
Precision treatment of colorectal cancer through radiolabeled antibodies	Pretargeted immunological Positron Emission Tomography (immuno-PET) using the following: an anti-carcinoembryonic antigen (CEA) recombinant bispecific monoclonal antibody (BsMAb); TF2 and the [68Ga]Ga-labeled HSG peptide IMP288 in patients with metastatic colorectal carcinoma (CRC).	This technology allows enhanced imaging accuracy and targeted treatment approaches, thereby minimizing harm to surrounding healthy tissue.	Potential side effects, such as allergic reactions, infusion-related complications, and long-term immunosuppression, may increase the likelihood of infections.	[108]
Imaging application	Pretargeted immuno-PET using anti-CEA/anti-IMP288 BsMAb and a [68Ga]Ga-labeled hapten: safe and feasible. Promising diagnostic performance.	Provides a reliable and effective diagnostic approach, demonstrating encouraging precision in identifying tumor locations and tracking the spread of metastasis.	Possible adverse effects could encompass allergic reactions, symptoms associated with the infusion (such as fever and chills), and a likelihood of immune suppression, which may elevate the risk of infections.	[108]
Precision treatment of colorectal cancer through radiolabeled antibodies	Cysteine site-specific 89Zr-labeled anti-CD73 (89Zr-CD73) IgG immuno-PET technique: can image tumor CD73 expression in living bodies.	This aspect allows accurate visualization of tumor-related CD73 expression, enhancing the precision of diagnosis and monitoring in colorectal cancer.	Higher production costs and possible radiation exposure risks could make it hard to use on a larger scale; more clinical testing is needed to prove long-term safety and effectiveness.	[109]
Imaging application	89Zr-CD73 IgG: showed CD73-dependent specific binding to cancer cells; provided high-contrast PET imaging of CD73 expressing tumors; 89Zr-CD73 IgG PET may be useful for the non-invasive assessment of tumor CD73 expression in living subjects.	Delivers enhanced PET imaging of tumors that express CD73, facilitating the early identification and tracking of colorectal cancer metastases.	Radiation exposure hazards require specific facilities for manufacturing and meticulous management, as well as limited accessibility for broad clinical application.	[109]
177Lu-labeled CA19–9 monoclonal antibody via PET imaging for colorectal cancer therapy	A 177Lu-labeled CA19–9 monoclonal antibody was screened via PET imaging for colorectal cancer therapy.	Combines treatment and diagnostic operations, allowing for accurate targeting of cancer cells and immediate observation of treatment effectiveness.	There is a chance that radioactive substances will be harmful. Because of this, PET imaging facilities are specialized, and strict safety rules must be followed when handling radiolabeled antibodies.	[110]
Imaging application	Radiolabeled CA19–9 mAb for CRC treatment: both 89Zr-DFO-C003 for CRC immune-PET imaging and 177Lu-DOTA-C003 for radiotherapy against CRC exhibit good potential in clinical application.	It provides versatile applications for both imaging and treatment, effectively targeting CRC cells.	It requires advanced imaging technology and incorporates radioactive substances, potentially leading to safety issues and regulatory hurdles.	[110]

## Data Availability

No new data were created or analyzed in this study.
